# Editorial

**Published:** 1916-11

**Authors:** 


### EDITORIAL

The Atlanta Journal-Record of Medicine has its Business Office at 23 West Alabama Street where all business communications should be sent.

Remittances are payable to The Journal-Record of Medicine.

Reprints of Original Articles will be furnished at cost price. Requests for them had best be made on the Manuscript.

Upon request, we will present, postpaid, to each contributor of an original article, twenty copies of The Journal-Record of Medicine containing such article.

### IS SYPHILIS CURABLE?

Before the appearance of Salvarsan and the later methods of treating syphilis the patient with this disease was told that it would require two or three years to effect a cure. With the advent of Salvarsan the outlook for a much surer and quicker cure created a wave of optimism; however, this has been on the wane for some time and clinicians hesitate to pronounce the syphilitic cured under two or more years just as before the Salvarsan era.

However, it remained for *Warthin to show us the frequency of active syphilitic lesions in "cured" and latent cases. In the pathological service in the University of Michigan from 1912-1914, Warthin performed autopsies on forty-one cases which showed characteristic syphilitic lesions or the presence of spirochetes. These forty-one cases constituted one-third of the adult autopsies for this period. He has divided these cases into three classes: First, cases where there is a definite history of syphilis and where the patient has received adequate treatment and has been pronounced cured. Second, cases in which the syphilitic infection was recognized as being active and where the treatment was still being carried out. Third, cases where a history of syphilis could not be obtained and where the clinical diagnosis was not syphilis and no treatment had been given.

In eleven cases of the first group he was able to demonstrate spirochetes in every case, usually in the heart, aorta and testes. In the five cases under active antisyphilitic treatment spirochetes were demonstrated in one or more of the following: heart, aorta, testes, central nervous system. But of still more interest is the fact that he found active syphilitic lesions in twenty-five cases, nearly one-fifth of all adult autopsies from 1912-1914, where there was no history of syphilis and where the clinical diagnosis did not include syphilis. Another fact of interest and value is that in these twenty-five cases he found sixteen cases of subacute or chronic parenchymatous nephritis; two of acute parenchymatous nephritis and five cases of interstitial nephritis. However, he found no direct relationship between syphilis and the kidney lesions present.

Dr. Warthin says that in all these forty-one patients the lesions of active syphilis were of the same nature, that is, cases that had been "cured," cases that were being treated and those which had received no treatment at all. Active syphilitic lesions were found in the heart in thirty-six cases; in the aorta in thirty-two cases; in the tests in thirty-one cases; in the liver in four cases; in the adrenals in six cases; in the spleen in one case; in the pancreas in six cases; and the central nervous system in five cases. Another interesting point is that in all five of the active cases in class two, the central nervous system showed marked syphilitic changes. Added weight is given to this work by Warthin when it is recognized that he took as a criterion for diagnosis only typical spirochetes and did not depend merely upon the pathological changes.

In the face of these findings it would seem that syphilis is merely rendered latent in the great majority of cases and the

---

*Warthin, A. S. The Persistence of Active Lesions and Spirochetes in the Tissues of Clinically Inactive or "Cured" Syphilis, The American Journal of the American Journal of the Medical Sciences, Vol. CLII, No. 4, pg. 508. October, 1916.

number of cures is really very few. Are not a great many unexplained deaths following rather simple operations the result of latent syphilis? May this not explain the numbers of deaths following operations where the patient would naturally be expected to survice? Is it not true that the older clinicians were correct in advising their patients to return each year or two for a course of treatment after the "cure"? While we would not like to make the statement that "once a syphilitic always a syphilitic," we will say that the syphilitic individual should be watched carefully not for one or two years, but for a number of years and would add to *Nichols standard of cure which is: one year without treatment, without any suspicious clinical signs, with a number of negative Wassermann reactions and no positive ones and with a negative Provocative Wassermann and a negative leutin test at the end of the year; that in addition to these, the patient should show a normal spinal fluid and should be watched for three to five years, especially where the disease advanced as far as the secondary stage before treatment was begun.

---

*Nichols, H. J. Studies of Syphilis. Bulletin No. 3, War Department: office of the Surgeon General; June, 1913; page 129.

